# Soil Alveolata diversity in the undisturbed steppe and wheat agrocenoses under different tillage

**DOI:** 10.18699/VJGB-23-81

**Published:** 2023-10

**Authors:** N.B. Naumova, P.A. Barsukov, O.A. Baturina, M.R. Kabilov

**Affiliations:** Institute of Soil Science and Agrochemistry of the Siberian Branch of the Russian Academy of Sciences, Novosibirsk, Russia; Institute of Soil Science and Agrochemistry of the Siberian Branch of the Russian Academy of Sciences, Novosibirsk, Russia; Institute of Soil Science and Agrochemistry of the Siberian Branch of the Russian Academy of Sciences, Novosibirsk, Russia; Institute of Chemical Biology and Fundamental Medicine of the Siberian Branch of the Russian Academy of Sciences, Novosibirsk, Russia

**Keywords:** ITS region, ciliates, Chernozem, conventional tillage, no tillage, ITS район, инфузории, чернозем, традиционная вспашка, нулевая обработка

## Abstract

Microeukaryotes are vital for maintaining soil quality and ecosystem functioning, however, their communities are less studied than bacterial and fungal ones, especially by high throughput sequencing techniques. Alveolates are important members of soil microbial communities, being consumers and/or prey for other microorganisms. We studied alveolate diversity in soil under the undisturbed steppe (US) and cropped for wheat using two tillage practices (conventional, CT, and no-till, NT) by amplifying the ITS2 marker with ITS3_KYO2/ITS4 primers and sequencing amplicons using Illumina MiSeq. A total of 198 Alveolata OTUs were identified, with 158 OTUs attributed to the Ciliophora phylum, containing five classes: Litostomatea, Spirotrichea and Oligohymenophorea, Nassophorea and Phyllopharyngea. Litostomatea and Phyllopharyngea were more abundant in US as compared with CT and NT. The observed OTU richness was higher in US than in CT and NT. The β-biodiversity of soil ciliates also very distinctly differentiated the US field from CT and NT. In the US, Nassophorea and Spirotrichea correlated positively with sand and negatively with clay, silt and SOM contents. This is the first report about soil ciliates diversity in Siberia as assessed by metabarcoding technique. The revealed clear effect of land use on the relative abundance of some taxa and a lack of tillage effect suggest the importance of the quantity and quality of plant material input for shaping the prey for ciliates. The ITS-metabarcoding technique was used for the first time in the research of ciliates diversity; further studies, embracing diverse aspects of soil ciliates by combining -omics methodology with the traditional one, are needed to get a better insight on the ecological roles of the main ciliate taxa in the complex soil system.

## Abstract (ru)

Микроскопические эукариоты крайне важны для обеспечения качества почвы и функционирования экосистем. Однако сообщества почвенных микроэукариот менее изучены по сравнению с сообществами бактерий и грибов, особенно с применением методов высокопроизводительного секвенирования. Значимыми компонентами почвенных микробных сообществ являются альвеоляты, участвующие в ключевых процессах почвенных экосистем (разложение органического вещества, трансформация питательных элементов и др.). Цель работы заключалась в изучении разнообразия альвеолят в почве под ненарушенной степной растительностью и при возделывании пшеницы с помощью двух методов обработки почвы (традиционная вспашка и нулевая обработка) путем амплификации маркера ITS2 с праймерами ITS3_KYO2/ITS4 и последующего секвенирования ампликонов (Illumina MySeq). Всего идентифицировано 198 ОТЕ альвеолят, из которых 158 относились к типу Ciliophora и пяти его классам: Litostomatea, Spirotrichea, Oligohymenophorea, Nassophorea и Phyllopharyngea. Litostomatea и Phyllopharyngea оказались более обильны в почве под ненарушенной степной растительностью по сравненению с почвой под пшеницей обоих вариантов обработки. Богатство ОТЕ в верхнем слое ненарушенной почвы под степной растительностью было также заметно выше, чем в обоих вариатах возделываемых полей, которые четко отличались от степи и по β-биоразнообразию. Nassophorea и Spirotrichea положительно коррелировали с содержанием песка в ненарушенной и пахотной почве; в последней к ним присоединились Litostomatea. Данная работа представляет собой первое исследование разнообразия почвенных альвеолят с применением метода метабаркодирования. Выявленное воздействие землепользования на относительное обилие некоторых таксонов наряду с отсутствием влияния обработки почвы свидетельствует о важном значении количества и качества поступающего в почву растительного материала для формирования сообществ микроорганизмов, поедаемых инфузориями. Дальней- шие исследования с применением методолгии -омик и традиционных способов необходимы для лучшего понимания экологической роли инфузорий в частности и альвеолят в целом в сложной почвенной системе.

## Introduction

As it is currently argued, “bridging the gaps between biodiversity science and agricultural practices is crucial to meet food security in the Anthropocene” (Cappelli et al., 2022. P. 674). Therefore, belowground biodiversity is an ultimately important part/mediator of such efforts. Eukaryotic microorganisms, such as fungi, alveolates, metazoans, algae and other organisms with ≤ 5000 μm3 of body volume (Coleman, 1985) are important players in biotic interactions in soil, and, as such, involved in key ecosystem processes: organic matter transformation, nutrient cycling, etc. (Bardgett, Putten, 2014). Protozoa were shown to benefit plant growth (Bonkowski, 2004), for instance, by improving N mineralization from soil organic matter via stimulating bacterial biomass turnover (Kuikman et al., 1990). Thus, microeukaryotes presence is vital for maintaining soil quality and ecosystem functioning and sustainability. However, soil microbial eukaryotic communities are much less studied as compared with bacterial and fungal ones, and especially by the high throughput sequencing techniques. Alveolates are important members of soil microbial assemblages, where they serve as consumers or prey for other microorganisms. The abundance and taxonomic diversity of alveolates used to be studied by culturing (by the so-called most probable numbers technique) and microscopy. Currently microscopy is the main methodology for the enumeration of alveolates (Adl et al., 2008), but species identification, requiring a complicated staining protocol (Acosta-Mercado, Lynn, 2003), is rather laborious and sometimes not definitive. Therefore, metagenomic approach and state-of-the-art high throughput sequencing has greatly extended the methodology for assessing the biodiversity of alveolates in soil.

In agricultural ecosystems soil and its residential biota is strongly affected by all aspects of production technologies, such as tillage, fertilization, crops, pesticides and others. Over the last decades the possibility to reduce damage to soil by minimizing tillage has gained much attention from both researchers and practitioners. Although there are many reports about bacterial and fungal biodiversity estimated metagenomically under minimal and/or no tillage, alveolates have remained relatively understudied (Ritter et al., 2021). While assessing the ITS2 region DNA sequence reads diversity, using the ITS3_KYO2/ITS4 primer set (Liu K. et al., 2012), under different tillage practices in the chernozem in the south of West Siberia (Naumova et al., 2022), we found that those fungal primers also amplified DNA belonging to other domains, specifically Alveolata, Amoebozoa, Heterolobosea, Metazoa, Rhizaria and Eukaryota kingdoms of uncertain taxonomic attribution. All those reads were discarded for the mycobiome analysis (Naumova et al., 2022); however, a substantial number of alveolate operational taxonomic units (OTUs), with their rarefaction curves reaching plateau with increasing number of sequence reads, convinced us to proceed with analyzing alveolate ITS-based diversity.

## Materials and methods

Experimental site and conditions. The field trial was described earlier (Naumova et al., 2022) (https://www.mdpi. com/2075-1729/12/8/1169). Briefly, the study area is the forest-steppe zone (54°4′6″ N, 79°36′3″ E) with a sharply continental climate1, the mean monthly temperature in the area of experimental site location in October is 3.5 °C with Luvic Endocalcic Chernozem (Siltic) (World Reference Base for Soil Resources…, 2015) as the widely spread and agriculturally significant soil of the region.


Supplementary Materials are available in the online version of the paper: https://meteoinfo.ru/en/ climate/monthly-climate-means-for-towns-of-russia-temperature-andprecipitation (accessed on March 27, 2022).


Experimental setup. The field trail was described earlier as well (Naumova et al., 2022). Briefly, it was started in 2009 on the area of 40 ha when a portion of the conventionally tilled soil (CT, mouldboard ploughing in the fall and disking in the spring) was subjected to the no-till technology (NT); both plots were getting the same rates of herbicides and fertilizers simultaneously.

The wheat grain yield, harvested at the beginning of September 2021, reached 4.8 t ha–1 in the NT field and 4.1 t ha–1 in the CT field. An undisturbed site (Un), located near the experimental field and covered by a true bunchgrass steppe (with Stipa capillata, Festuca valesiaca, some Poa spp. and Puccinellia sp.), was used to get the data about the zonal soil bacteriobiome as a reference.

Soil sampling and chemical analyses. Soil was sampled in October 2021 from the 0–5 and 5–15 cm layers in five individual replicates from each layer. In total, 30 soil samples were collected and chemically analyzed as described before (Naumova et al., 2022): soil pH ranged 6.3–6.8, total soil carbon content ranged 3.6–4.2 %, and total soil nitrogen content was 0.29–0.37 %.

DNA extraction, amplification and sequencing. Total DNA was extracted with the DNeasy PowerSoil Kit (Qiagen, Hilden, Germany) according to the manufacturer’s instructions. The bead-beating was performed using TissueLyser II (Qiagen, Hilden, Germany) for 10 min at 30 Hz. Agarose gel electrophoresis was used to assess the quality of the extracted DNA; additional DNA purification was not necessary

The ITS2 gene marker was amplified with the primer pairs ITS3_KYO2/ITS4, combined with Illumina adapter sequences (Fadrosh et al., 2014). PCR amplification was performed as described earlier (Kryukov et al., 2020). A total of 200 ng PCR product from each sample was pooled together and purified using the MinElute Gel Extraction Kit (Qiagen, Hilden, Germany). The obtained amplicon libraries were sequenced with 2x300 bp paired-ends reagents on MiSeq (Illumina, CA, USA) in the SB RAS Genomics Core Facility (ICBFM SB RAS, Novosibirsk, Russia). The read data reported in this study were submitted to the NCBI Short Read Archive under bioproject accession number PRJNA845814.

Bioinformatic analysis. Raw sequences were analyzed with the UPARSE pipeline (Edgar, 2013) using Usearch v.11.0.667. The UPARSE pipeline included merging paired reads; read quality filtering (-fastq_maxee_rate 0.005); length trimming (remove less 100 nt); merging identical reads (dereplication); discarding singleton reads; removing chimeras and OTU clustering using the UPARSE-OTU algorithm. The OTU sequences were assigned a taxonomy using SINTAX (Edgar, 2013) and ITS UNITE USEARCH/UTAX v.8.3 (Abarenkov et al., 2021) as a reference. Taxonomic structure of sequences thus obtained was estimated by the ratio of the number of taxon-specific sequence reads (with non-fungal removed from the data matrix) to the total number of sequence reads, i. e. by the relative abundance of taxa, expressed as percentage.

The OTUs datasets were analyzed by individual rarefaction with the help of the PAST software (Hammer et al., 2001): the number of alveolate OTUs detected, reaching plateau with increasing number of sequences, showed that the sampling effort was close to saturation for all samples, thus being enough for comparing biodiversity (Hughes, Hellmann, 2005).

Statistical analyses. Statistical analyses (descriptive statistics, ANOVA and correlation analyses) were performed by using Statistica v.13.3 (TIBCO Software Inc., Palo Alto, CA, USA). Rarefaction curves, OTUs-based α-diversity indices were calculated and principal coordinates analysis was performed using PAST software. Factor effects and mean differences in post-hoc comparisons by Fisher’s LSD test were considered statistically significant at the p ≤ 0.05 level.

## Results

**Fig. 1.**
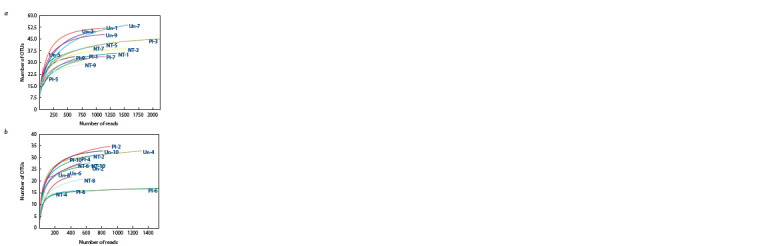
The rarefaction curves for alveolate OTUs in the 0–5 (a) and 5–15 (b) cm soil. Un – undisturbed soil, Pl – ploughed and NT – no-till soil; the numbers indicate individual soil replicates from a tillage treatment.

Alveolata taxonomic diversity. After quality filtering, chimera and other domains’ sequences removal, a total of 198 different Alveolata OTUs were identified at 97 % sequence identity level, with 158 OTUs attributed to the Ciliophora phylum, the rest remaining unclassified below the domain level. The rarefaction curves showed that the sampling effort was enough to compare diversity (Hughes, Hellmann, 2005), as the number of OTUs dependent on the total number of sequence reads reached plateau (Fig. 1).

Five Ciliophora classes were identified: Litostomatea with 32 OTUs being the most OTU-rich one, followed by Spirotrichea and Oligohymenophorea with 25 OTUs each, Nassophorea and Phyllopharyngea being represented by just three OTUs each. Of the total number of Ciliophora OTUs, many (70, or 44 %) remained unassigned to the lower taxonomic levels.

Taxonomic composition and structure in different fields. The relative abundance of the Ciliophora phylum did not differ between the fields and the layers (Table 1), whereas at the class level there were some differences: Litostomatea was much more abundant in the undisturbed soil as compared to both cropped ones, and Phyllopharyngea, albeit being a minor member of the ciliate assemblage, was also markedly increased in the undisturbed soil. At the order level, Sporadotrichida was almost seven times more abundant in the 0–5 cm layer of the undisturbed soil than in the no-till one. Oligohymenophorea_is, an order-level cluster, had almost five times higher abundance in the 5–15 cm layer of the no-till soil as compared with the undisturbed one (Table 1). Haptorida (Litostomatea), Hymenostomatida (Oligohymenophorea) and Cyrtophorida (Phyllopharyngea) displayed much higher abundance in the undisturbed soil.

**Table 1.**
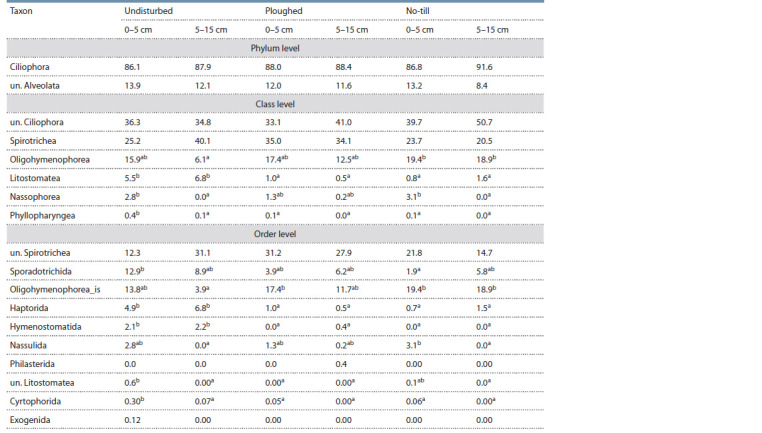
Relative abundance (%, mean) of the dominant Alveolata taxa in Chernozem 0–5 and 5–15 cm layers in the experimental fields in the south of West Siberia Note.. “un.” stands for unclassified. Letters in rows indicate that the values are different ( p ≤ 0.05, Fisher’s LSD test); the absence of letters after the values in a row indicates that there was no difference.

**Fig. 2.**
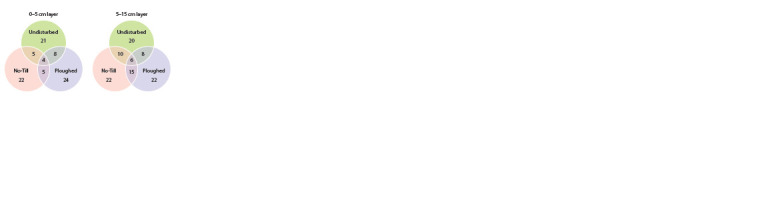
Venn’s diagram of the number of the dominant Alveolata OTUs in soil under different tillage treatment. OTUs were considered dominant if they accounted for ≥ 1 % of the total number of sequence reads.

The number of dominant OTUs slightly exceeded 20 in each field and both layers (Fig. 2). The sets, bulked over both soil layers, comprised 30–43 dominant OTUs, the number of the dominant OTUs being maximal in the undisturbed soil. Three OTUs were common for all fields, with two OTUs not identified below the class level (Spirotrichea) and one OTU, below the phylum level (Ciliophora). As for the 0–5 cm layer, the fourth dominant OTU belonged to the Nassulida order of the Nassophorea class; in the 5–15 cm layer, in addition to the three OTU-level clusters common for all samples, there were two unidentified ones below the phylum level and one was identified to the Spirotrichida order of the Spirotrichea class.

Alpha- and beta-biodiversity in different fields. The observed OTU richness was notably higher in the 0–5 cm layer of the undisturbed soil (Table 2) as compared with both cropped ones; the cropped soils did not differ from each other in this index. As for the potential OTU richness, though its estimator (Chao1) in the 0–5 cm layer of the undisturbed soil was markedly higher than in the respective layer of the CT soil, the same was true, albeit to a lesser extent, for the respective layer of the NT soil. Shannon index was much increased in the 0–5 cm layer of the undisturbed soil as compared with its 5–15 cm layer, displaying no difference between the fields.

**Table 2.**
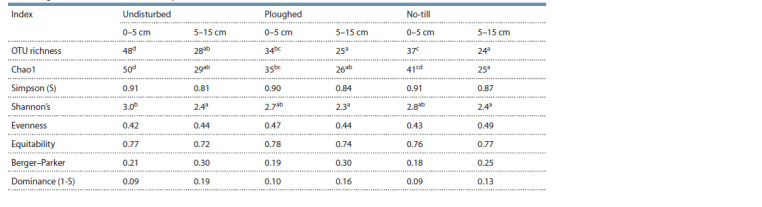
Alpha-biodiversity indices (calculated on the OTU’s basis) of Alveolata OTUs assemblages in the Chernozem in the experimental fields in the south of West Siberia Note. Letters in rows indicate that the values are different ( p ≤ 0.05, Fisher’s LSD test); the absence of letters after the values in a row indicates that there was no difference

**Fig.3.**
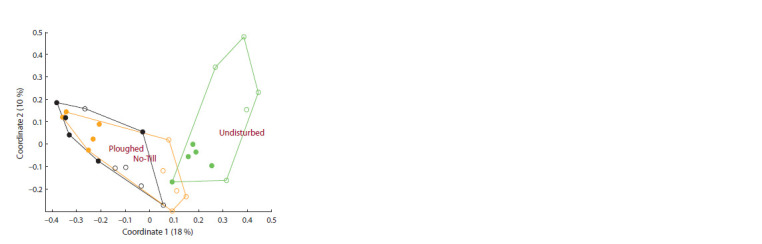
Principal coordinates analysis of the soil alveolate assemblage composition (OTU level, Bray–Curtis dissimilarity distance) under different soil tillage in the forest-steppe zone in West Siberia: location of samples in the plane of the first two coordinates Solid circles indicate samples from the 0–5 cm layer, and open circles indicate samples from the 5–15 cm layer.

As for β-diversity, it clearly separated the undisturbed field from the cropped ones, and the latter were not separated from each other (Fig. 3).

**Fig. 4.**
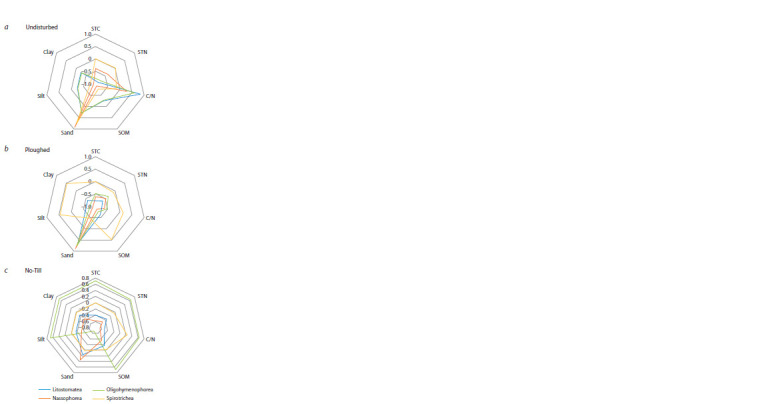
Correlation coefficients (Pearson) between the relative abundance of the dominant ciliate classes in 0–5 cm layer and soil properties: STC, STN – soil total carbon and nitrogen, SOM – soil organic matter, C/N – the STC/STN ratio in soil; undisturbed soil (a), ploughed soil (b) and no-tillage soil (c). The coefficients |r| ≥ 0.88 were statistically significant at p ≤ 0.05 level.

Ciliate assemblage composition and soil properties. Correlation of the ciliate classes abundance with soil properties in the 0–5 cm layer showed specific spectra for each field (Fig. 4), albeit with few correlation coefficients being statistically significant.

In the undisturbed soil, Nassophorea and Spirotrichea correlated positively with sand and negatively with clay, silt and soil organic matter contents, being also sensitive to pH. In the same soil, Litostomatea and Oligohymenophorea showed preference for soil organic matter with a wide C/N ratio. The pattern was different in the CT soil, where all major classes, except for Spirotrichea, correlated positively with sand and negatively with clay, silt and soil organic matter ones. A different correlation pattern was revealed in the NT soil, where Oligohymenophorea correlated positively with silt, clay, soil organic matter, soil total carbon and nitrogen and negatively with sand.

## Discussion

To assess the diversity of microscopic eukaryotes, most studies used primers to 18S rRNA genes (Ritter et al., 2021), either taxonomically broad (Chaib De Mares et al., 2017) or specific for soil ciliates (Lara et al., 2007; Ting et al., 2015). However, even with specific primers, non-target taxa sequences are commonly amplified (Pastorelli et al., 2022). The ITS primers we used in this study were designed for fungi (Liu K. et al., 2012), but often these primers did not fail to amplify a plethora of other domains, such as Alveolata, Amoebozoa, Heterolobosea, Metazoa, Rhizaria and Eukaryota kingdoms of uncertain taxonomic attribution. That was precisely what happened in our research: we analysed and reported the mycobiome data (Naumova et al., 2022), but, besides fungal sequences, obtained many sequence clusters belonging to other domains, including Alveolata. It seemed a waste not to attempt an analysis of such sequences; the more so as the number of OTUs reached plateau with the increasing number of sequence reads, tempting us to compare diversity of amplicon sequences. The primers we used were not specifically designed for alveolates, and we are far from claiming that we examined alveolate assemblages in their entirety (though even with specific primers, such claims would be unjustified). However, the fact that diversity estimates of Alveolata OTUs showed agronomically and ecologically meaningful differences, together with our humble hope that our study would “clearly benefit from incorporating more protistology alongside the study of bacteria, fungi and animals” (Geisen et al., 2018), strongly encouraged us to discuss how ciliate diversity relates to the context of this study.

Averaged over all samples in the study, Ciliophora accounted for 88 % of the Alveolata sequence reads. As Ciliophora were shown to prefer arid and semi-arid soil environments (Bates et al., 2013), the phylum ultimate dominance in Alveolata assemblage complies with the climate of the study region, characterized as semi-arid. Less Ciliophora presence, i. e. 45 % of the total number of sequence reads, was reported for the meadow soils in the Alps (Seppey et al., 2020). However, ciliates are relatively more studied and better represented in various databases: in particular, they are highly overrepresented in molecular surveys because of their shorter SSU rRNA sequences that ease amplification, and the presence of extremely high SSU rRNA gene copy numbers (Gong et al., 2013), ranging from 103 to 106 (Wang et al., 2019). All these might have also been a factor contributing to higher Ciliophora presence in our study.

This study explicitly identified five class-level sequence reads clusters, i. e. Spirotrichea, Oligohymenophorea, Litostomatea, Nassophorea and Phyllopharyngea. The first four were the dominant ones, being commonly found in other studies (employing the same or different methodology) as the main members of soil ciliate assemblages (and even estuarine ones (Jiang et al., 2021)). In the meadow soils in the Alps, the Ciliophora phylum was mostly represented by the Spirotrichea, Oligohymenophorea, Litostomatea and Colpodea classes (Seppey et al., 2020). In soil ciliate community at the Baiyun Mountain in China, the most species-rich classes were Spirotrichea, Colpodea, Litostomatea, Oligohymenophorea, Nassophorea, Armophorea and Phyllopharyngea, as determined by microscopy (Li et al., 2010); exactly the same class-level composition (by DGGE + sequencing) was reported for the oil palm plantation in Malaysia (Ting et al., 2012). Another study reported the ultimate (55 %) dominance of colpodids (by classical methodology) (Bamforth, 2001) in a range of different soils; a notable presence of the group (found by metatransciptomics) was also reported (Geisen et al., 2015). Another very recent study of ciliates in Castanozems in the north-west of China identified nine classes of soil ciliates (Liu H. et al., 2022), with Spirotrichea and Litostomatea being the most species-rich and Colpodea ranking third. Unlike all those studies, here, we did not find any Colpodea. This notable and surprising discrepancy concerning the Colpodea presence may be explained by differences in methodology, primers used, ecosystems, soil, as well as weather conditions preceding soil sampling. According to BLAST (https://blast. ncbi.nlm.nih.gov/Blast.cgi), the primers used for this study had poor homology for Colpodea. As for the weather conditions, they could have contributed somewhat as well: we collected soil samples at the end of the growing season with air temperatures dropping to negatives at night, but in the Alps soil samples were collected in July, i. e. at the height of the growing season, whereas at the Baiyun Mountain they were collected seasonally throughout a year. Also, as Colpodea are well known as r-strategists (Lüftenegger et al., 1985), e. g. thriving in unstable environments, they could hardly display a notable presence after gradual change of environmental conditions at the very end of the growing season, more than a month after harvest in the cropped fields and no disturbance in all three fields. Although there exists a discrepancy between the morphological and molecular databases used in the molecular barcoding of protists (Venter et al., 2018), the factor could have hardly contributed to the absence of such conspicuous and well-studied taxon as Colpodea in our study. Therefore, the primers are the primary culprits, and this difference between the ciliate classes might be worth looking into.

Our finding that the Litostomatea class was markedly increased in the undisturbed soil, as compared with the both cropped ones, suggests that in the undisturbed soil a) there was much more prey available for these free-living predators of other protists or microscopic animals (Vd’ačný et al., 2012), or b) these ciliates or their prey were susceptible to the agronomic practices in the cropped fields, as protists were shown to be the most susceptible soil microbiome component to the application of nitrogen fertilizers (Zhao Z. et al., 2019). Anyway, the undisturbed soil environment in both layers apparently benefited Litostomatea, perhaps indirectly, via different chemical composition/stoichiometry of the versatile plant litter: nutrient characteristics of the latter were shown to be important ecological factors that affect protozoan community diversity (Jia et al., 2021). And our finding of positive Litostomatea correlation with the wider C/N ratio of soil indirectly corroborates this.

The Spirotrichea representatives are commonly found in various soil environments, ranging from the high Arctic deserts (Choe et al., 2021) to meadows in the Alps (Seppey et al., 2020). Our results showing that it was the most abundant class in all soil samples from undisturbed and cropped fields (averaging 30 %) imply increased availability of their various prey, from bacteria to other ciliates (Subphylum 2. INTRAMACRONUCLEATA…, 2010), stimulated by dead phytomass abundance at the end of the growing season. The Spirotrichea diversity was reported to be positively correlated with physicochemical parameters such as interstitial water, total organic carbon, nitrogen and phosphorous content (Abraham et al., 2019). However, within the context of our study, i. e. small gradients of physicochemical properties, we did not reveal such a correlation at the wheat-cropped sites, but at the undisturbed site, the relative abundance of the Spirotricheaspecific sequences was found to correlate positively with sand content in soil. Such correlation suggests sensitivity of these ciliates to soil pore space and aeration, or both.

The fact that Oligohymenophorea had a substantial presence in all soil samples in our study (15 % on average) agrees with the notion that the class is a common member of soil ciliate assemblages (Zhao F. et al., 2013; Tribun et al., 2022). The finding that the class did not show any tillage-related differences (in the top layer) may suggest a broad spectrum of the class niches within the context of the study. As for the 5–15 cm layer, the Oligohymenophorea relative abundance increased from the undisturbed site to the ploughed and no-till ones: the results suggest the beneficial effect of some environmental property (unaccounted in the study) in the no-till soil.

As for the Nassophorea representatives, in this study, the class presence seemed to benefit from the unploughed soil environment similarly in both the undisturbed and no-tillage 0–5 cm layers, which indicates mostly the effect of soil properties, rather than plant species and phytomass. Although Phyllopharyngea demonstrated layer-related differential abundance, as the class was the rare member of the soil ciliate community at all sites, we would not attempt to draw any ecological inference from the finding.

It is noteworthy that in both soil layers only few OTUs were common for all three fields, belonging to the Spirotrichea class and not being explicitly attributed to any of its orders.

Our finding that ciliate relative abundance was in most cases positively correlated with sand content in the soil indicates the importance of relatively bigger pore space needed for these microscopic eukaryotes as typically they are longer than 50 μm in body length (Lynn, 2017).

Temperature is an important factor controlling the ciliate community (Oshima et al., 2020) and affects the structure and functions of the soil microbial food web. For instance, ciliates can be destroyed by freezing temperatures, especially at soil moisture content exceeding 30 % (Müller et al., 2010). Some researchers suggested that “internally governed encystment may be an essential adaptation to an unpredictable environment in which individual protozoa cannot sense when the soil will dry out and will survive desiccation only if they have encysted in time” (Ekelund et al., 2002. P. 1096), or, extending the statement, when the soil will freeze or experience other adverse condition. In general, soil helps to preserve ciliate cysts in a viable state. Since the soil for our study was sampled at the end of October when freezing temperatures, at least at nights, are common, it is most likely that the diversity profile reflects the diversity of viable but non-active organisms.

It should be noted that relationships between ribotypic and phenotypic traits of protists across their life cycle stages remain largely unknown: recently, encystment and temperature were shown to influence intraindividual sequence polymorphisms of rDNA and rRNA (Zou et al., 2021). Thus, the rDNA copy number may affect the composition and structure of soil ciliate assemblages.

Nowadays, it is commonplace to reiterate that “conventional agricultural production systems… reduce soil biodiversity” (Harkes et al., 2019); and our finding of reduced ciliate OTUs’ richness, both observed and potential, in the top 5-cm layer of conventionally ploughed soil as compared with the undisturbed one, agrees with the statement. However, the same was true for the no-till soil as far as the observed OTUs’ richness is concerned. But the potential richness, i. e. the Chao1 index, albeit being somewhat lower still, did not differ statistically from the value in the undisturbed soil, very likely indicating the ongoing, albeit slowly, process of ciliate assemblage diversification due to the no-till treatment. As for the Shannon index, it showed no difference between the soil tillage managements. The same pattern, i. e. reduced OTUs’ richness under conventional tillage as compared with the undisturbed soil, and no difference in the Shannon index, was found by us in the mycobiome of the same soil samples (Naumova et al., 2022). This finding implies that the Shannon index, calculated on the basis of sequence reads, in the case of eukaryotic microorganisms, at least such as fungi and alveolates, cannot adequately reflect biodiversity changes.

With ITS primers, this study recorded 158 Ciliophora OTUs in the soil samples collected from adjacent fields. Recently, a comprehensive study of ciliated protozoans in soils and fresh water bodies of the Russian Far East, performed by employing traditional microscopic techniques to detect and identify ciliates, found 307 species (Tribun et al., 2022), which, bearing in mind the number, biotope diversity and area surveyed (in total, about 900,000 km2) did not strike us as seriously exceeding the species richness in our study. The core of the ciliate communities in both studies belonged to the classes Oligohymenophorea, Spirotrichea and Litostomatea, together accounting for 65 % of species richness in the Far East study and 52 % of species richness and 41–50 % of the total number of sequence reads in our study. Another very recent study in the north-west of China, also employing traditional methodology to identify and enumerate ciliates, found 114 species of ciliates among four sampling sites, varying in vegetation and land use (Liu H. et al., 2022). Thus, we can safely conclude that soil ciliates diversity data, obtained here by sequencing amplicons of ITS2 region of rRNA genes, encompassed a significant portion of true ciliate diversity in soil, providing ecologically relevant and meaningful assemblage profiles in the context of our study.

## Conclusion

This is the first report about soil ciliates diversity, as assessed by metagenomic technique, in Siberia, and specifically in Chernozem under different land use and tillage practices (undisturbed steppe vs. cropped for wheat by conventional or no tillage). We found a clear effect of land use on the relative abundance of some taxa at the order level, but did not find any effect of the tillage treatments: this strongly suggests the importance of primary producers, i. e. the quantity and quality of plant material input in soil, in shaping the prey available for ciliates. Soil ciliate β-diversity differentiated the undisturbed field from the cropped ones very distinctly as well. Further multifaceted studies, focusing on many aspects of soil ciliates by combining -omics methodology with the traditional one, are needed to get a better insight on the ecological roles of the main ciliate taxa in the complex soil system.

## Conflict of interest

The authors declare no conflict of interest.
